# Enhanced Exopolysaccharide Production in Bidirectional Liquid Fermentation of *Ganoderma lucidum* Using *Clinacanthus nutans* (Burm. f.) Lindau

**DOI:** 10.3390/microorganisms14030624

**Published:** 2026-03-10

**Authors:** Zhen Chen, Shupei Zhang, Zimeng Wang, Pengru Li, Wanying Du, Jialu Li, Dan Chen, Mengyuan Yang, Kexin Zheng, Peng Yang, Xiaoyu Wei, Andong Gong

**Affiliations:** 1 Henan Key Laboratory of Tea Plant Biology, College of Tea and Food Science, Xinyang Normal University, Xinyang 464000, China; chenzhen@xynu.edu.cn (Z.C.); 17627018632@163.com (S.Z.); wzm19561752860@163.com (Z.W.); pr2107815947@163.com (P.L.); zhengkx202510@163.com (K.Z.); fwjt63298@126.com (P.Y.); 2 Dabie Mountain Laboratory, Xinyang 464000, China; 3 College of Life Science, Xinyang Normal University, Xinyang 464000, China; 18437507687@163.com (W.D.); 15503752782@163.com (J.L.); 18738359219@163.com (D.C.); 17539565798@163.com (M.Y.); 4 Technical Innovation Center for Utilization of Marine Biological Resources, Third Institute of Oceanography, Ministry of Natural Resources, Xiamen 361005, China

**Keywords:** *Ganoderma lucidum*, exopolysaccharides, bidirectional liquid fermentation, *Clinacanthus nutans*, antioxidant activity

## Abstract

This study explored the enhancement of exopolysaccharide (EPS) production by *Ganoderma lucidum* through bidirectional liquid fermentation, employing *Clinacanthus nutans* (Burm. f.) Lindau leaves as a medicinal substrate. The optimal concentration of *C. nutans* leaf powder was determined to be 6 g/L, resulting in a significant increase in both mycelial biomass (61.78%) and EPS yield (116.6%). Structural analyses indicated that the EPS supplemented with *C. nutans* underwent notable modifications. Fourier-transform infrared spectroscopy suggested the introduction of potential carbonyl groups and a shift in glycosidic linkage configuration. Monosaccharide composition analysis revealed a significant transition from a glucose-dominated profile in the control to a galactose-rich, more diverse profile, including uronic acids and amino sugars, in the experimental group. High-performance gel permeation chromatography demonstrated a transformation from a low, homogeneous molecular weight (4.7 kDa) to a heterogeneous, bimodal distribution featuring a prominent high-molecular-weight fraction (38.5 kDa). Consequently, the modified EPS exhibited significantly enhanced antioxidant activities, with scavenging rates for DPPH, hydroxyl, and ABTS radicals increasing to 55.5%, 35.1%, and 88.0%, respectively, at a concentration of 2 mg/mL. These findings demonstrate that *C. nutans* is an effective supplement for modulating the fermentation process of *G. lucidum*, not only boosting EPS production but also tailoring its structural characteristics to obtain polysaccharides with superior bioactivities, highlighting its potential in functional food and nutraceutical applications.

## 1. Introduction

*Ganoderma lucidum*, a renowned medicinal mushroom, has been used for centuries in traditional medicine across Asia [1]. Modern research attributes its extensive health-promoting properties—including immunomodulation, antitumor, antioxidant, and hypoglycemic effects—primarily to its bioactive polysaccharides [2]. The global market for *G. lucidum* products is substantial and growing, underscoring the high demand for these bioactive compounds [3]. While artificial cultivation of fruiting bodies is possible, it is limited by a lengthy growth cycle and batch-to-batch variability. In contrast, submerged liquid fermentation for producing exopolysaccharide (EPS) offers a promising alternative, featuring a shorter production cycle, superior controllability, and scalability [4]. However, the yield and bioactivity of EPS are highly dependent on fermentation conditions, driving continuous research into optimized strategies [5,6].

Traditional optimization focuses on physicochemical parameters. For instance, controlling fermentation pH at 4.0 was shown to increase *G. lucidum* EPS yield by 24% [7], while optimizing carbon and nitrogen sources in a starch-based medium doubled EPS concentration [4]. Beyond yield, the molecular weight (MW) of polysaccharides is a critical determinant of bioactivity. Recent innovations in directed fermentation aim to produce high-MW polysaccharides (>1000 kDa), which are associated with more potent antioxidant and immunomodulatory activities [8]. In this context, bidirectional liquid fermentation has emerged as a sophisticated strategy to enhance and tailor fungal metabolite production. This approach involves cultivating medicinal fungi with specific plant substrates [9]. The substrate acts not only as a nutrient source but also as a biostimulant, potentially inducing metabolic reprogramming in the fungus. This can lead to the synthesis of novel or structurally modified compounds with enhanced bioactivity [10]. A practical example is the addition of astragalus (*Astragalus membranaceus*) to *G. lucidum* culture, which significantly increased EPS yield [11]. This demonstrates the potential of plant–fungal interactions to improve fermentation outcomes.

*Clinacanthus nutans* (Burm. f.) Lindau, commonly known as Sabah Snake Grass, is a traditional medicinal plant with documented anti-inflammatory, antiviral, and potent antioxidant properties [12]. Its leaves are particularly rich in bioactive phytochemicals, including flavonoids (e.g., schaftoside, vitexin, orientin), polyphenols, chlorophyll derivatives, and polysaccharides [13]. Beyond their direct pharmacological activities, these compounds have been reported to function as elicitors in fungal fermentation systems. For instance, flavonoids such as naringenin and quercetin have been shown to stimulate polysaccharide production in various medicinal fungi, including *Cordyceps militaris* and *Inonotus obliquus* [14,15]. Similarly, plant-derived oligosaccharides have been reported to act as elicitors in fungal systems, modulating secondary metabolism and enhancing the production of bioactive metabolites [16]. Based on these premises, we hypothesize that incorporating *C. nutans* leaf powder into the *G. lucidum* fermentation medium serves a dual function: (i) Nutritional enhancement: The leaf powder provides supplementary carbon, nitrogen, minerals, and vitamins that directly support increased mycelial growth and primary metabolism, thereby boosting overall EPS yield. (ii) Metabolic modulation: Specific phytochemicals (particularly flavonoids and phenolic acids) act as elicitors that bind to putative fungal membrane receptors, activating signal transduction pathways. This elicitor signaling is expected to reprogram the expression of genes involved in nucleotide sugar biosynthesis and glycosyltransferase activity, redirecting carbon flux from simple glucan synthesis toward the assembly of a structurally complex heteropolysaccharide enriched in galactose and uronic acids. Furthermore, plant-derived monosaccharides or oligosaccharides released during fermentation may be taken up by the fungus and directly incorporated into the sugar nucleotide precursor pool, contributing to the altered monosaccharide profile. Therefore, this study aims to systematically evaluate the impact of *C. nutans* on the bidirectional fermentation of *G. lucidum*. By establishing a causal link between plant substrate addition, fungal metabolic response, and polysaccharide functionality, this work seeks to provide a comprehensive strategy for the efficient production of high-value, functionally tailored EPS for nutraceutical applications.

## 2. Materials and Methods

### 2.1. Chemicals and Reagents

Glucose, peptone, yeast extract, MgSO_4_·7H_2_O, KH_2_PO_4_, vitamin B1, NaCl, and ethanol were purchased from Sinopharm Chemical Reagent Co., Ltd. (Shanghai, China). Trifluoroacetic acid (TFA, ≥99%), phenol, sulfuric acid, potassium bromide (KBr, FT-IR grade), DPPH (1,1-diphenyl-2-picrylhydrazyl), ABTS (2,2′-azino-bis(3-ethylbenzothiazoline-6-sulfonic acid)), potassium persulfate, FeSO_4_, H_2_O_2_, salicylic acid, and ascorbic acid (vitamin C) were obtained from Sigma-Aldrich (St. Louis, MO, USA). Monosaccharide standards (fucose, rhamnose, arabinose, galactose, glucose, xylose, mannose, fructose, ribose, galacturonic acid, glucuronic acid, glucosamine hydrochloride, N-acetyl-D-glucosamine, guluronic acid, and mannuronic acid) were supplied by Borui Sugar Biotech Co., Ltd. (Shenzhen, China). Dextran standards for molecular weight calibration (Mp: 6.9, 10.6, 21.8, 46.5, 110.0, 218.5, 409.0, and 782.7 kDa) were purchased from Showa Denko (Tokyo, Japan). All chemicals were of analytical grade or higher.

### 2.2. EPS Production

#### 2.2.1. Strain and Culture Conditions

The *G. lucidum* strain CZ01 is preserved in our laboratory and routinely maintained on potato dextrose agar (PDA) slants. For the preparation of the seed culture, fresh mycelia of *G. lucidum* were transferred from PDA plates to 250 mL conical flasks containing 50 mL of seed medium. The seed medium composition was as follows: 30 g/L glucose, 3 g/L yeast extract, 2 g/L MgSO_4_·7H_2_O, 2 g/L KH_2_PO_4_, and 0.01 g/L vitamin B. The cultures were incubated at 28 °C with agitation at 150 rpm in a shaker for a duration of 7 days. Subsequently, the mycelial aggregates were disrupted to produce a mycelium suspension using a cell disruptor. This suspension was then inoculated at a concentration of 2% (*w*/*v*) into 50 mL of fermentation medium and incubated under the same conditions (28 °C, 150 rpm) for 7 to 10 days. The fermentation medium was composed of 40 g/L glucose, 4 g/L peptone, 1.5 g/L MgSO_4_·7H_2_O, 1.5 g/L KH_2_PO_4_, and 0.01 g/L vitamin B. To identify the optimal medicinal substrate for bidirectional liquid fermentation, 5 g/L of dry leaf powder (100 mesh) from Vernonia amygdalina Del., *C. nutans* (Burm. f.) Lindau, and *Orthosiphon aristatus* (Blume) Miq. was incorporated into the fermentation medium. To identify the optimal addition concentration, the leaf powder of *C. nutans* at concentrations of 2, 4, 6, 8, and 10 g/L was added to the fermentation medium. To verify that the EPS originated solely from fungal fermentation and not from direct leaching of the plant material, a non-inoculated control was prepared: a fermentation medium containing 6 g/L *C. nutans* leaf powder was incubated under identical conditions (28 °C, 150 rpm) without *G. lucidum* inoculation.

#### 2.2.2. Assessment of Biomass and EPS Production

The mycelia were isolated from the fermentation broth through vacuum filtration. Subsequently, the mycelia were rinsed with distilled water and subjected to freeze-drying until a constant weight was achieved for the purpose of biomass quantification. The fermentation broth, devoid of mycelia, underwent centrifugation at 12,000 *g* for 10 min at 4 °C to eliminate insoluble particulates. The resultant supernatant was subjected to extensive dialysis for 24 h at 4 °C using a dialysis membrane with a molecular weight cut-off of 1000 Da. Following dialysis, the retentate was concentrated under reduced pressure at 40 °C to approximately one-tenth of its original volume. Three volumes of pre-chilled absolute ethanol were added, and the mixture was kept at 4 °C overnight to precipitate the exopolysaccharides. The precipitate was collected by centrifugation (12,000 *g*, 20 min, 4 °C), re-dissolved in a minimal amount of deionized water, and freeze-dried for 48 h to a constant weight. This combined dialysis–ethanol precipitation protocol effectively removes low-molecular-weight contaminants (MW < 1000 Da) and enriches high-molecular-weight polysaccharides, yielding a crude EPS preparation suitable for structural characterization and bioactivity screening.

### 2.3. Compositional Analysis of EPS

The protein content of EPS preparations was determined using the Bradford method [17]. Briefly, 1 mL of Bradford reagent was added to 100 μL of EPS solution (1 mg/mL in deionized water). After 5 min incubation at room temperature, the absorbance was measured at 595 nm. Bovine serum albumin (BSA) was used as a standard to construct a calibration curve (0–100 μg/mL). Total sugar content was quantified by the phenol–sulfuric acid method [18] using glucose as the standard.

### 2.4. Structural Characterization of EPS

#### 2.4.1. Infrared Spectral Analysis of EPS

The functional groups present in EPS were analyzed using Fourier Transform Infrared (FT-IR) spectroscopy [19]. A precise measurement of 2 mg of the sample was combined with 200 mg of KBr, and the mixture was compressed into tablets. A blank control was prepared by compressing pure KBr powder into tablets. Both the sample and the blank control were placed separately in the FT-IR650 spectrometer (manufactured by Tianjin Gangdong Technology Development Co., Ltd., Tianjin, China) for scanning and recording. The spectral analysis was conducted over a range of 4000 to 400 cm^−1^.

#### 2.4.2. Determination of Monosaccharide Components in EPS

In accordance with Yang’s research methodology [20], with minor modifications, this study employed ion chromatography (IC) to ascertain the monosaccharide composition. Sixteen monosaccharide standards, each weighing 5 mg—comprising fucose, rhamnose, arabinose, galactose, glucose, xylose, mannose, fructose, ribose, galacturonic acid, glucuronic acid, glucosamine hydrochloride, N-acetyl-D-glucosamine, guluronic acid, and mannuronic acid—were placed in ampoules. Subsequently, 2 mL of 3 M TFA was added and the samples were hydrolyzed at 120 °C for 3 h. The resulting acid hydrolysate was precisely extracted and transferred to a tube for nitrogen evaporation and drying. The dried residue was then reconstituted in 5 mL of water, vortexed, and mixed thoroughly to prepare a standard stock solution. Precisely prepared concentration standards of the various monosaccharide solutions were utilized as mixed standards. Using an absolute quantitative approach, the mass of different monosaccharides was determined, and their molar ratios were calculated based on their respective molar masses. Precisely measure 5 mg of the freeze-dried sample and place it into an ampoule. Introduce 2 mL of 3 M TFA and conduct hydrolysis at 120 °C for a duration of 3 h. Carefully transfer the resulting acid hydrolysis solution into a tube and evaporate it to dryness using a nitrogen stream. Subsequently, add 5 mL of water, vortex, and mix thoroughly. Withdraw 50 µL of the sample and dilute it with 950 µL of deionized water. Centrifuge the mixture at 12,000 rpm for 5 min and collect the supernatant for ion chromatography analysis. The chromatography is performed using a Dionex Carbopac™ PA20 column (3 × 150 mm). The mobile phase consists of A: H_2_O; B: 15 mM NaOH; C: 15 mM NaOH and 100 mM NaOAc, with a flow rate of 0.3 mL/min. The injection volume is set at 5 µL, and the column temperature is maintained at 30 °C. Detection is carried out using an electrochemical detector.

#### 2.4.3. Determination of the Molecular Weight of EPS

The molecular weight distribution of EPS was determined using high-performance gel permeation chromatography (HPGPC) [21]. The mobile phase was 0.5 M NaCl solution, filtered through a 0.22 μm membrane and degassed. Chromatographic separation was performed on a BRT105-103-101 series gel column (8 × 300 mm; three columns in tandem) maintained at 40 °C, with a flow rate of 0.7 mL/min and an injection volume of 25 μL. Detection was carried out using a refractive index detector (RID-20A, Shimadzu, Tokyo, Japan). Calibration: Eight dextran standards with peak molecular weights (Mp) of 6.9, 10.6, 21.8, 46.5, 110.0, 218.5, 409.0, and 782.7 kDa (Showa Denko, Tokyo, Japan) were dissolved in mobile phase (5 mg/mL) and analyzed in triplicate. Calibration curves were constructed by plotting the logarithm of molecular weight (LgMp, LgMw, LgMn) against retention time (RT). The derived equations were as follows: LgMp-RT: y = −0.1807x + 11.251 (R^2^ = 0.9991); LgMw-RT: y = −0.1795x + 11.204 (R^2^ = 0.9994); LgMn-RT: y = −0.1802x + 11.217 (R^2^ = 0.9990). Sample solutions (5 mg/mL in mobile phase) were centrifuged (12,000 rpm, 10 min), filtered (0.22 μm), and analyzed under identical conditions. Molecular weights (Mp, Mw, Mn) were calculated by substituting the retention time of each peak into the respective calibration equations. The polydispersity index (PDI = Mw/Mn) was calculated for each peak to assess the breadth of the molecular weight distribution.

### 2.5. Antioxidant Activity Assays of EPS

#### 2.5.1. ·OH Free Radical Scavenging Activity

The assessment of ·OH free radical scavenging activity was conducted with slight modifications to the method described by Xu et al. [22]. A series of EPS sample solutions were prepared at concentrations of 0.25, 0.5, 1.0, 1.5, and 2.0 g/L using deionized water. To each 1 mL sample solution, 2 mL of 9 mmol/L FeSO_4_ and 2 mL of 9 mmol/L H_2_O_2_ were added, thoroughly mixed, and allowed to react at room temperature for 10 min. Subsequently, 2 mL of 9 mmol/L C_7_H_6_O_3_ was added, and the reaction was continued for an additional 10 min at room temperature. The absorbance was measured at a wavelength of 510 nm (denoted as A_s_). For the sample control group, deionized water was used in place of H_2_O_2_ to measure absorbance (A_j_), while the blank group used deionized water instead of the sample solution to measure absorbance (A_0_). Vitamin C served as the positive control. The ·OH free radical scavenging activity was calculated using the following formula: ·OH free radical scavenging activity (%) = [(A_0_ − (A_s_ − A_j_))/A_0_] × 100%.

#### 2.5.2. DPPH Free Radical Scavenging Activity

The assessment of DPPH free radical scavenging activity was conducted following the method outlined by Ma et al. [23], with minor modifications. EPS sample solutions were prepared at concentrations of 0.25, 0.5, 1.0, 1.5, and 2.0 g/L using deionized water. Subsequently, 50 μL of each sample solution was transferred to a 96-well plate, followed by the addition of 150 μL of a 0.05 mmol/L DPPH solution (prepared in methanol to a concentration of 0.05 mM). The mixtures were incubated at room temperature, shielded from light, and thoroughly mixed for 30 min. The absorbance was then measured at a wavelength of 517 nm (denoted as A_s_). For the sample control group, the absorbance (A_j_) was measured using methanol in place of the DPPH solution, while for the blank group, the absorbance (A_0_) was measured using deionized water instead of the sample solution, with vitamin C serving as the positive control. The DPPH radical scavenging activity was calculated using the following formula: DPPH free radical scavenging activity (%) = [(A_0_ − (A_s_ − A_j_))/A_0_] × 100%.

#### 2.5.3. ABTS Free Radical Scavenging Activity

The assessment of ABTS free radical scavenging activity follows the methodology outlined by Liao et al. [24], with minor modifications. To begin, prepare 1 mL of EPS sample solutions at concentrations of 0.25, 0.5, 1.0, 1.5, and 2.0 g/L using deionized water. Prepare a 7 mmol/L ABTS solution and mix it with an equal volume of 1.4 mmol/L potassium persulfate. Allow this mixture to stand overnight in the dark at room temperature. Prior to use, dilute the solution with distilled water until the absorbance at 734 nm reaches 0.7 ± 0.02. Subsequently, mix 100 μL of the EPS solution with 100 μL of the ABTS solution and allow the reaction to proceed at room temperature for 5 min. Measure the absorbance (A_s_) at 734 nm. For the sample control group, replace the ABTS solution with distilled water and measure the absorbance (A_j_). In the blank group, substitute the sample solution with deionized water and measure the absorbance (A_0_), using vitamin C as the positive control. The formula for calculating the ABTS free radical scavenging rate is as follows: ABTS free radical scavenging activity (%) = [(A_0_ − (A_s_ − A_j_))/A_0_] × 100%.

### 2.6. Statistical Analysis

Each experiment was conducted in triplicate and subjected to statistical analysis utilizing the OriginPro 2024 (Version 10.1.0.157). Data are expressed as mean ± standard deviation (SD). Statistical significance was assessed using Student’s *t*-test, with a *p*-value of less than 0.05 deemed statistically significant.

## 3. Results

### 3.1. Screening of Medicinal Substrate for EPS Production by G. lucidum

**Figure 1:**
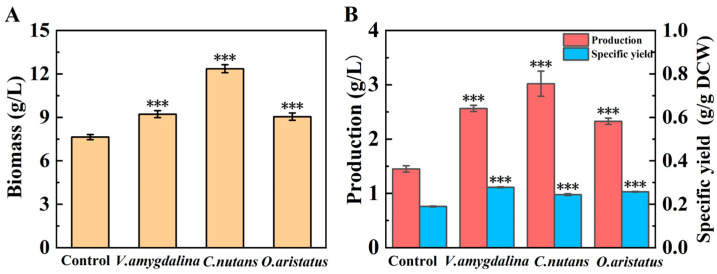
Effect of various medicinal substrates on mycelial growth (**A**) and EPS production (**B**). All data are expressed as mean ± SD of three independent experiments. ***: *p* < 0.001.

To identify suitable medicinal substrates for enhancing EPS production during the fermentation of *G*. *lucidum*, various substrates, including *V. amygdalina* Del., *C. nutans* (Burm. f.) Lindau, and *O. aristatus* (Blume) Miq., were incorporated into the fermentation medium. As illustrated in Figure 1A, the addition of these medicinal substrates significantly increased the mycelial biomass. Notably, *C*. *nutans* exhibited the most pronounced effect, elevating the mycelial biomass to 12.36 g/L, which represents an increase of 61.78%. Correspondingly, EPS production was also enhanced (Figure 1B), aligning with the observed increase in mycelial biomass. Furthermore, the specific yield of EPS increased from 0.19 to 0.24 g/g DCW. These findings indicate that the enhancement of EPS production is linked not only to improved mycelial growth but also to an increased capacity for EPS synthesis. The results demonstrate that the supplementation of medicinal substrates, particularly *C*. *nutans*, effectively promotes both the growth of *G*. *lucidum* and its capacity for EPS synthesis. Consequently, *C. nutans* was selected as the optimal medicinal substrate for EPS production by *G. lucidum*.

### 3.2. Mycelial Growth and EPS Production Under Different Concentrations of C. nutans Leaves

**Figure 2:**
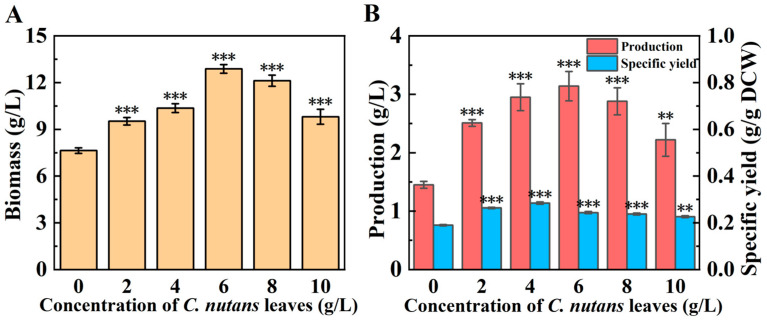
Effect of concentration of *C. nutans* leaves on mycelial growth (**A**) and EPS production (**B**). All data are expressed as mean ± SD of three independent experiments. **: *p* < 0.01; ***: *p* < 0.001.

The effects of *C. nutans* leaf powder with different concentrations on the growth and EPS production of *G. lucidum* are shown in Figure 2. The results indicate that the incorporation of *C. nutans* leaf powder markedly enhanced the biomass of *G. lucidum*, with the dry cell weight peaking at a powder concentration of 6 g/L. Beyond this concentration, a further increase in the powder addition resulted in a decline in dry cell weight. Similarly, EPS production exhibited an initial increase followed by a decrease as the concentration of *C. nutans* leaf powder was augmented. The maximum EPS production, recorded at 3.14 g/L, occurred at a 6 g/L powder concentration, representing a 116.60% increase compared to the control group. Consequently, the optimal concentration of *C. nutans* leaf powder for EPS production was determined to be 6 g/L. The non-inoculated control yielded no detectable polysaccharide precipitate after ethanol addition, and the freeze-dried product was negligible (weight < 0.1 mg from 50 mL culture). The phenol–sulfuric acid assay confirmed the absence of measurable total sugar. These findings unequivocally demonstrate that the EPS obtained in our study is exclusively produced by *G. lucidum* during fermentation and is not derived from direct leaching of polysaccharides or other macromolecules from the *C. nutans* leaves.

### 3.3. Compositional Analysis of EPS Preparations

To assess the purity of the isolated EPS, the protein and total sugar contents were determined (Table 1). The control EPS contained 2.34 ± 0.21% protein and 92.4 ± 2.8% total sugar, while the experimental EPS contained 3.18 ± 0.27% protein and 90.9 ± 3.2% total sugar. These values indicate that the dialysis–ethanol precipitation protocol effectively removed most soluble proteins and low-molecular-weight contaminants, yielding EPS preparations with high polysaccharide purity (>90%).

### 3.4. Effect of C. nutans Leaves on Functional Groups of EPS

**Figure 3:**
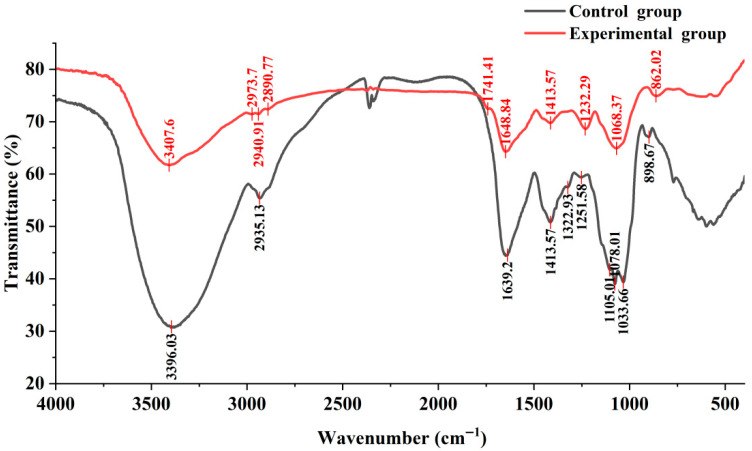
Comparison of infrared spectrometer of the EPS between the control and experimental groups.

FT-IR spectroscopy was employed to investigate the influence of *C. nutans* leaves on the functional groups of EPS produced by *G. lucidum* during liquid fermentation, as illustrated in Figure 3. The FT-IR spectra of EPS from both the experimental group (with 6 g/L *C. nutans* leaves powder) and the control group (without *C. nutans* leaves powder) exhibited characteristic polysaccharide absorption bands. Common features included broad O–H stretching vibrations (experimental: 3407 cm^−1^; control: 3396 cm^−1^) and C–H stretching vibrations (experimental: 2973, 2940, 2890 cm^−1^; control: 2935 cm^−1^), confirming the polysaccharide nature of the samples [19]. However, notable differences were observed between the two groups. The experimental group showed an additional absorption peak at 1741 cm^−1^, which may be attributed to C=O stretching vibrations, possibly indicating the presence of carboxyl or ester groups not prominent in the control. The C=O stretching band was also observed at 1648 cm^−1^ in the experimental group and 1639 cm^−1^ in the control [25]. Furthermore, the fingerprint region revealed structural variations: the experimental group displayed a peak at 862 cm^−1^, characteristic of α-configuration in pyranose rings, while the control exhibited a peak at 898 cm^−1^, typical of β-configuration [26]. These shifts suggest that the addition of *C. nutans* leaves powder may alter the glycosidic linkage configuration or introduce structural modifications in the EPS. Absorption bands related to C–O stretching and O–H bending were present in both groups but with slight wavenumber variations. In conclusion, the incorporation of *C. nutans* powder into the fermentation medium appears to influence the functional group profile of EPS, particularly introducing potential carbonyl groups and affecting the anomeric configuration of sugar rings.

### 3.5. Effect of C. nutans Leaves on Monosaccharide Components in EPS

**Figure 4:**
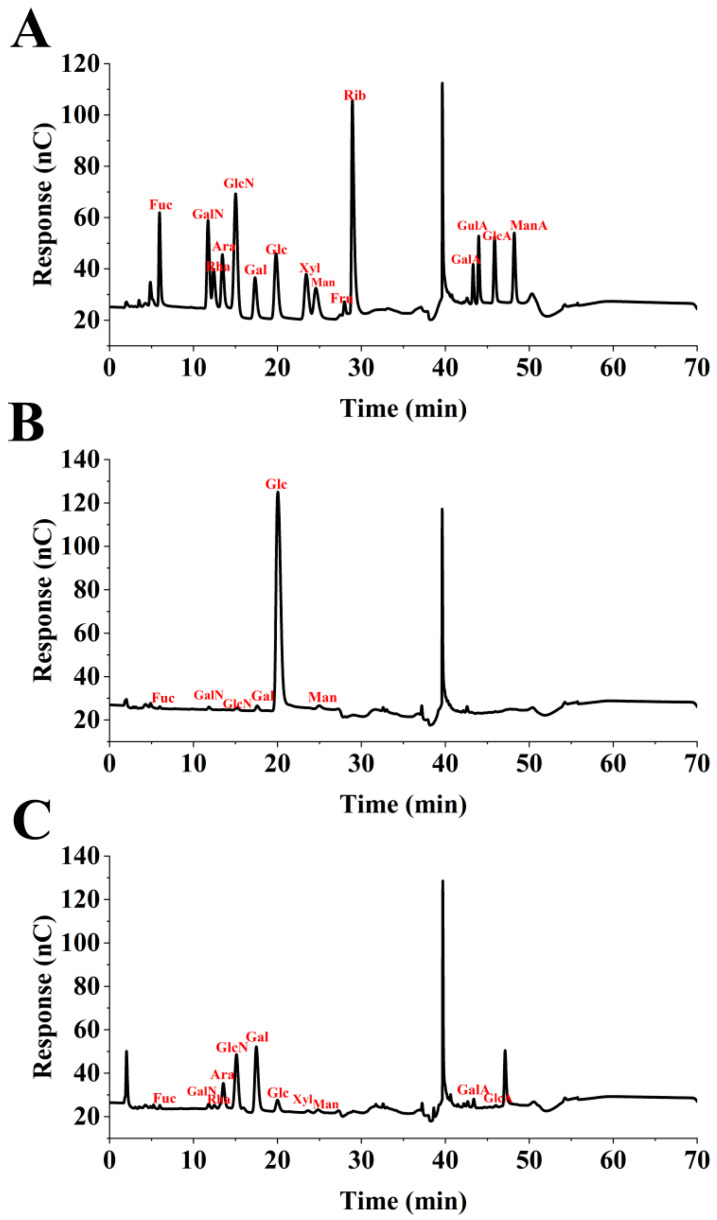
Ion chromatogram of standard monosaccharide (**A**); EPS monosaccharide of the control group (**B**); EPS monosaccharide of the experimental group (**C**).

The influence of *C. nutans* leaves on the monosaccharide composition of EPS produced by *G. lucidum* during liquid fermentation was determined using ion chromatography (IC) analysis, as illustrated in Figure 4 and Table 2. Significant differences were observed between the experimental and control groups. The EPS from the experimental group contained a more diverse array of monosaccharides, including fucose, galactosamine hydrochloride, rhamnose, arabinose, glucosamine hydrochloride, galactose, glucose, xylose, mannose, galacturonic acid, and glucuronic acid. In contrast, the EPS from the control group primarily consisted of fucose, galactosamine hydrochloride, glucosamine hydrochloride, galactose, glucose, and mannose. Notably, the molar ratios of key monosaccharides differed substantially between the two groups. In the experimental group, galactose was the predominant monosaccharide (molar ratio: 0.556), followed by glucose (0.055). Conversely, in the control group, glucose was the most abundant monosaccharide (molar ratio: 0.936), while galactose was present in a much lower proportion (0.027). The findings suggest that incorporating *C. nutans* leaves into the fermentation medium not only enhances the diversity of monosaccharide components in the EPS but also significantly modifies their relative abundances. This compositional shift, notably characterized by a substantial increase in galactose and a corresponding decrease in glucose, implies that *C. nutans* leaves may influence the metabolic pathways of *G. lucidum*. This modulation could result in the biosynthesis of an EPS with a unique and potentially more intricate glycosidic structure, which may subsequently affect its physicochemical properties and bioactivities.

### 3.6. Effect of C. nutans Leaves on Molecular Weight of EPS

**Figure 5:**
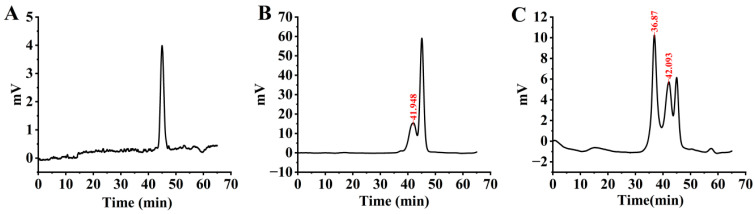
High-performance liquid gel permeation chromatography of the blank phase (**A**); EPS of the control group (**B**); EPS of the experimental group (**C**). Mn is the number average molecular weight; Mp is the peak position molecular weight; Mw is the weight average molecular weight.

The effect of *C*. *nutans* leaves on the molecular weight distribution of EPS produced by *G*. *lucidum* was analyzed using high-performance gel permeation chromatography (HPGPC). As illustrated in Figure 5 and Table 3, the results revealed a significant impact. The EPS from the control group exhibited a single, symmetric peak with a retention time (RT) of 41.948 min, corresponding to a relatively low and homogeneous molecular weight (Mw of approximately 4724 Da). In stark contrast, the EPS from the experimental group displayed a bimodal distribution. It consisted of two distinct fractions: a major high-molecular-weight fraction (RT 36.870 min, Mw ~38,533 Da, 58.8% of the total peak area) and a minor low-molecular-weight fraction (RT 42.093 min, Mw ~4449 Da, 41.2% of the total peak area). This indicates that the addition of *C. nutans* leaves to the fermentation medium fundamentally altered the polymerization process, leading to the biosynthesis of EPS with a much broader and heterogeneous molecular weight profile. This substantial increase in molecular weight, coupled with the observed heterogeneity, could profoundly influence the viscosity, solubility, and, ultimately, biological activities of the produced EPS.

### 3.7. Effect of C. nutans Leaves on Antioxidant Activity of EPS

**Figure 6:**
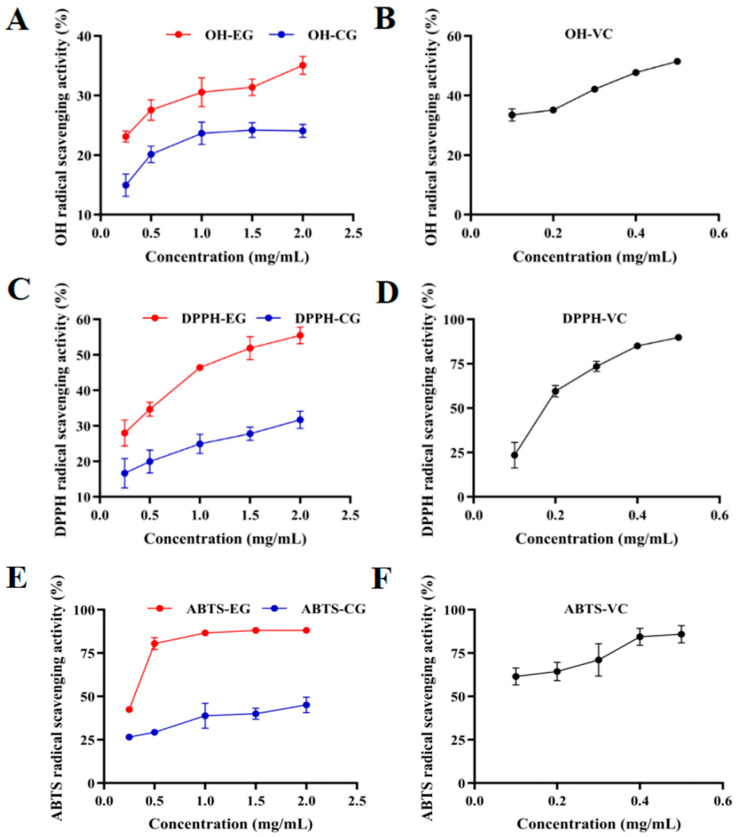
Evaluation of the antioxidant activity of the EPS. Scavenging activity of ·OH free radicals by various concentration of EPS and (**A**) and vitamin C (**B**); scavenging activity of DPPH free radicals by various concentration of EPS (**C**) and vitamin C (**D**); scavenging activity of ABTS free radicals by various concentration of EPS (**E**) and vitamin C (**F**). CG: control group EPS (fermented without *C. nutans*); EG: experimental group EPS (fermented with 6 g/L *C. nutans* leaf powder); VC: vitamin C (positive control).

The antioxidant activity of the EPS produced by *G*. *lucidum* was significantly influenced by the addition of *C*. *nutans* leaves to the fermentation medium. As assessed by three standard in vitro assays (DPPH, hydroxyl radical, and ABTS radical scavenging) (Figure 6), the EPS from the experimental group consistently exhibited superior radical scavenging capacity compared to the control group across all tested concentrations (0.25–2.0 mg/mL). At the highest concentration of 2 mg/mL, the scavenging rates of the Experimental groups were 55.5% (DPPH), 35.1% (·OH), and 88.0% (ABTS), which were markedly higher than those of the Control groups at 31.7%, 24.1%, and 45.1%, respectively. This pronounced enhancement in antioxidant potency can be attributed to the structural modifications induced by *C. nutans* leaves, as revealed by previous analyses. The polysaccharides of the experimental group possessed a more diverse monosaccharide profile, including higher proportions of potential antioxidant contributors such as uronic acids (galacturonic acid, glucuronic acid) and amino sugars. Furthermore, the polysaccharides of the experimental group featured a distinct high-molecular-weight fraction (~38.5 kDa), which is often associated with improved bioactivity. The synergistic effect of this altered chemical composition—potentially providing more active hydroxyl, carboxyl, and other functional groups for electron donation—and the presence of larger polymer chains likely underpin the significantly strengthened free radical neutralization capability. These findings demonstrate that *C. nutans* leaves is an effective fermentation supplement not only for modulating the yield and structure of *G. lucidum* EPS but also for strategically enhancing its functional antioxidant properties, which are valuable for potential applications in nutraceuticals or functional foods.

## 4. Discussion

The present study demonstrates that supplementation of the *G. lucidum* fermentation medium with *C. nutans* leaf powder at an optimal concentration of 6 g/L significantly enhances both mycelial biomass (61.78% increase) and EPS yield (116.6% increase). The observed yield enhancement aligns with previous reports on plant-based fermentation enhancers. Zhou et al. [27] reported that the addition of coixenolide significantly improved polysaccharide production in submerged cultures of *G. lucidum*, while Liu et al. [28] demonstrated that *Vernonia amygdalina* leaf powder enhanced exopolysaccharide production in *Inonotus hispidus*. These studies collectively support the premise that medicinal plant substrates can provide essential nutrients and growth factors that upregulate primary metabolism and key enzymes in polysaccharide biosynthetic pathways. The mechanistic basis for this enhancement likely involves multiple factors. First, *C. nutans* leaves are rich in carbohydrates, proteins, and minerals that may serve as supplementary nutrients. Second, and more importantly, the bioactive phytochemicals present in *C. nutans*—including flavonoids, phenolic acids, and terpenoids—may function as elicitors that activate fungal secondary metabolism. Similar elicitor mechanisms have been documented in fungal–plant interactions; for instance, Li et al. [29] demonstrated that elicitors derived from *Aspergillus niger* significantly upregulated the expression of genes involved in bioactive compound biosynthesis in *Glycyrrhiza uralensis* adventitious roots, including cinnamate 4-hydroxylase, *β*-amyrin synthase, squalene epoxidase, and cytochrome P450 monooxygenase. This provides a precedent for exogenous elicitors modulating secondary metabolism through gene expression regulation. Wang et al. [9] further established a bidirectional fermentation system using *Monascus* and mulberry leaves, demonstrating that this approach significantly increased bioactive content and promoted secondary metabolism, with RT-qPCR analysis confirming upregulation of key biosynthetic genes at the transcriptional level.

Beyond yield enhancement, the most significant finding of this work is the profound structural reprogramming of EPS induced by *C. nutans*. FT-IR analysis revealed the emergence of a distinct C=O stretching peak at 1741 cm^−1^ in the supplemented EPS (Figure 3), which is characteristic of uronic acid carbonyl or *O*-acetyl groups [25]. This spectroscopic evidence was robustly corroborated by monosaccharide analysis, which detected galacturonic and glucuronic acids exclusively in the experimental group (Table 1). Uronic acids are pivotal for polysaccharide bioactivity; their carboxyl groups enhance molecular hydrophilicity and provide metal-chelating sites, which are directly linked to improved antioxidant and immunomodulatory capacities. This structure–function relationship is strongly supported by prior research; Yang et al. [30] directly demonstrated that an increase in uronic acid content of *G. lucidum* EPS correlates closely with enhanced antioxidant activity against DPPH radicals. He et al. [31] also showed that in polysaccharides from *Polyporus umbellatus*, those containing higher uronic acid content exhibited better antioxidant activity, suggesting that uronic acid residues play an important role in physiological functions. The shift in anomeric region absorption from 898 cm^−1^ (control) to 862 cm^−1^ (experimental) suggests a change in the dominant glycosidic linkage configuration from *β*-type to *α*-type [26]. Such configurational shifts can profoundly alter polysaccharide chain conformation, solubility, and recognition by biological receptors, thereby influencing functional properties. This is consistent with established principles in carbohydrate chemistry, where anomeric configuration is a key determinant of molecular flexibility and specific binding interactions with proteins, as discussed by Buley and Striegel [32]. Liao et al. [33] demonstrated that in five different acidic polysaccharides from Dendrobium, samples with *β*-configuration exhibited higher antioxidant activity than those with *α*-configuration, further supporting the potential bioactivity implications of the configurational shift observed in our study. Alias et al. [34] comprehensively reviewed that the mitigation of oxidative stress by polysaccharides is closely linked to structural features such as uronic acid content, degree of polymerization, and glycosidic configuration, providing mechanistic insight into their structure–activity relationships.

The transformation in monosaccharide composition was particularly striking. Control EPS exhibited a typical glucan-like profile, predominantly composed of glucose (93.6 mol%). In contrast, *C. nutans*-derived EPS was transformed into a complex heteropolysaccharide with galactose as the predominant monomer (55.6 mol%), accompanied by a diverse array of neutral sugars (arabinose, xylose, rhamnose), amino sugars (galactosamine, glucosamine), and uronic acids (galacturonic acid, glucuronic acid) (Table 1). This dramatic shift indicates that *C. nutans* supplementation actively diverted or expanded the sugar nucleotide precursor pool in *G. lucidum*. Peng et al. [35] studied the effects of culture conditions on the monosaccharide composition of *G. lucidum* exopolysaccharide and on activities of related enzymes, demonstrating that manipulation of fermentation parameters can significantly alter both enzyme activities and resulting polysaccharide composition. The dramatic increase in galactose content (from 2.7 mol% to 55.6 mol%) strongly suggests upregulation of UDP-glucose-4-epimerase, which interconverts UDP-glucose and UDP-galactose. The non-inoculated control experiment confirmed that no detectable polysaccharide was recovered from the medium containing only *C. nutans* leaves, ruling out direct co-extraction of plant polysaccharides. However, metabolic recycling of plant-derived carbohydrates remains plausible: soluble polysaccharides or glycoproteins released from the leaf powder during fermentation may be partially hydrolyzed by fungal glycoside hydrolases, generating monosaccharides or oligosaccharides that enter the fungal sugar nucleotide pool and are re-utilized for EPS biosynthesis. Such recycling has been reported in other fungal–plant co-culture systems [28]. Regardless of their ultimate origin (de novo synthesis or recycled precursors), the incorporation of these diverse monosaccharides into the EPS polymer is mediated by the fungus and reflects a fundamental shift in its metabolic program induced by *C. nutans*.

Concurrent with compositional changes, the molecular weight distribution underwent a fundamental alteration. EPS shifted from a homogeneous low-Mw population (~4.7 kDa) to a bimodal distribution, featuring a significant high-Mw fraction (~38.5 kDa, 58.8% of total peak area) (Figure 5, Table 3). While the precise mechanism underlying this increased molecular weight remains to be elucidated, several non-exclusive hypotheses can be proposed based on current knowledge of fungal polysaccharide biosynthesis: (i) enhanced processivity of glycosyltransferase complexes, (ii) primer-mediated elongation initiated by plant-derived oligosaccharides, or (iii) reduced hydrolytic cleavage by extracellular glucanases. The low polydispersity (PDI ≈ 1.03–1.04) indices for both fractions suggest regulated biosynthesis rather than random polymerization. Importantly, higher molecular weight in polysaccharides is frequently correlated with superior bioactivity, as longer chains can form more stable tertiary structures and present a greater density of active functional groups. Ma et al. [36] systematically demonstrated that *G. lucidum* polysaccharides at different growth stages exhibited varying molecular weights (112 kDa to 11,358 kDa) and that higher molecular weight fractions showed superior antioxidant activities, with the highest total antioxidant activity (18.79 mmol/mL) and DPPH radical scavenging capacity (36.15%) observed at the spore stage where molecular weight reached 7678 kDa. Their study also revealed that during maturation of *G. lucidum*, hexokinase and phosphoglucose isomerase activities showed increasing trends that correlated with changes in polysaccharide content and molecular weight, providing evidence for the link between sugar metabolism enzyme activities and final polysaccharide characteristics. Future work employing transcriptomics, targeted enzyme assays, and pulse-chase labeling experiments will be required to uncover the precise biochemical mechanism.

The significantly enhanced antioxidant activity—evidenced by scavenging rates of 55.5% (DPPH), 35.1% (·OH), and 88.0% (ABTS) at 2 mg/mL (Figure 6)—represents a 1.75- to 1.95-fold increase compared to the control EPS. Compositional analysis confirmed that both EPS preparations consist primarily of polysaccharides (>84%), with only minor protein contamination (<3.5%) and a negligible difference between groups (0.84%). These findings effectively exclude residual protein as a major contributor to the observed activity enhancement. Instead, the enhanced antioxidant capacity can be directly attributed to the specific structural modifications induced by *C. nutans* supplementation. The introduced uronic acids (14.1 μg/mg galacturonic acid and 1.54 μg/mg glucuronic acid in experimental EPS vs. undetectable in control) provide additional proton-donating sites for radical neutralization [31,34]. The complex heteropolymeric structure exposes more reactive hydroxyl groups, and the appearance of a high-molecular-weight fraction (38.5 kDa) facilitates effective radical quenching through extended electron delocalization [36]. The robust ABTS scavenging activity, in particular, underscores the potent electron-donating ability of this modified EPS, a property strongly linked to its unique structural signature [37]. These structure–activity correlations strongly suggest that the observed increase in antioxidant capacity is primarily attributable to the polysaccharide fraction itself.

In summary, this study demonstrates that *C. nutans* functions not only as a nutrient source that enhances yield but also as a significant metabolic modulator for *G. lucidum*. It effectively reconfigures the fungal biosynthetic pathways to generate a structurally unique, high-value heteropolysaccharide with enhanced antioxidant properties. This research highlights the potential of bidirectional fermentation as a strategic approach for the sustainable and targeted production of fungal polysaccharides for nutraceutical and pharmaceutical applications. Future investigations should aim to (1) elucidate the specific metabolic regulatory mechanisms activated by *C. nutans* through transcriptomic and metabolomic analyses, with a particular focus on genes encoding UDP-glucose-4-epimerase, glycosyltransferases, and sugar nucleotide biosynthetic enzymes; (2) incorporate advanced EPS purification steps (e.g., ion-exchange chromatography, size-exclusion chromatography) to isolate and characterize the specific polysaccharide sub-fractions responsible for enhanced bioactivity; and (3) assess the promising immunomodulatory and anti-tumor potential of the structurally refined EPS in appropriate in vitro and in vivo models.

## Figures and Tables

**Table 1:** Protein and total sugar content of EPS preparations.

Sample	Protein Content (%)	Total Sugar Content (%)
Control group	2.34 ± 0.21	92.4 ± 2.8
Experimental group	3.18 ± 0.27	90.9 ± 3.2

**Table 2:** Comparison of monosaccharide components between the control and experimental groups.

Name	Control Group			Experimental Group		
	Peak Area	Retention Time (min)	Molar Ratio	Peak Area	Retention Time (min)	Molar Ratio
Fuc	0.194 ± 0.015	5.992	0.005 ± 0.001	0.348 ± 0.027	5.992	0.011 ± 0.002
GalN	0.406 ± 0.032	11.859	0.005 ± 0.001	0.729 ± 0.058	11.809	0.013 ± 0.002
Rha	0	12.409	0	0.572 ± 0.045	12.484	0.025 ± 0.003
Ara	0	13.442	0	4.612 ± 0.368	13.55	0.107 ± 0.009
GlcN	0.529 ± 0.042	15.175	0.004 ± 0.001	11.687 ± 0.935	15.125	0.11 ± 0.009
Gal	1.014 ± 0.088	17.592	0.027 ± 0.003	15.423 ± 1.205	17.484	0.556 ± 0.041
Glc	65.038 ± 4.924	20.05	0.936 ± 0.068	2.835 ± 0.211	20.009	0.055 ± 0.005
Xyl	0	23.434	0	0.478 ± 0.038	23.642	0.015 ± 0.002
Man	0.974 ± 0.078	25.009	0.023 ± 0.002	0.841 ± 0.067	24.834	0.027 ± 0.003
GalA	0	43.309	0	0.917 ± 0.073	43.392	0.072 ± 0.006
GlcA	0	45.867	0	0.228 ± 0.018	46	0.008 ± 0.001

**Table 3:** Comparison of molecular weight between the control and experimental groups.

Sample	Retention Time (min)	Mp	Mw	Mn	PDI (Mw/Mn)	Peak Area (%)
Control group	41.948 ± 0.103	4688 ± 152	4724 ± 168	4550 ± 141	1.04 ± 0.02	100
Experimental group	36.87 ± 0.087	38778 ± 1245	38533 ± 1310	37413 ± 1228	1.03 ± 0.02	58.805 ± 3.2
	42.093 ± 0.112	4414 ± 178	4449 ± 185	4284 ± 169	1.04 ± 0.02	41.195 ± 3.2

## Data Availability

The original contributions presented in this study are included in the article. Further inquiries can be directed to the corresponding authors.

## Abbreviations

The following abbreviations are used in this manuscript:

EPS	Exopolysaccharide
MW	Molecular weight
PDA	Potato dextrose agar
FT-IR	Fourier Transform Infrared
KBr	Potassium bromide
DPPH	1,1-Diphenyl-2-picrylhydrazyl
ABTS	2,2′-Azino-bis(3-ethylbenzothiazoline-6-sulfonic acid)
RT	Retention time
IC	Ion chromatography
TFA	Trifluoroacetic acid
HAGPC	High-performance liquid gel permeation chromatography
